# Diseases of the Muscular Walls of the Heart

**Published:** 1872-07

**Authors:** Richard Quain

**Affiliations:** Physician to the Hospital for the Diseases of the Chest, Brompton


*Diseases of the Muscular Walls of the Heart.* Lectures delivered at the Royal College of Physicians, March, 1872, by RICHARD QUAIN, M.D., F.R.S., Physician to the Hospital for the Diseases of the Chest, Brompton.

In the attention which has been given to diseases of the valves of the heart, the affections of its walls have too much been lost sight of. True, their importance has not escaped the attention of the principal writers on the subject. Laennec, Stokes, Gairdner,

Chambers, Peacock, have all referred to the importance of studying the condition of the walls of the heart; but such prominence is still given to the subject of valvular disease that many persons have come to regard the term as synonymous with all heart disease, while there are not a few who regard dilatation and hypertrophy as mere complications of diseases of the valves. No doubt disease of the valves is a very serious element in the production of heart disease; it more or less inevitably leads to dilatation or dilated hypertrophy. But many other causes are equally efficient in producing those conditions. The real value of clinical study of the valvular diseases of the heart, is that a knowledge of their existence leads us to a recognition of the immediate cause of the disease which may be present in the heart's walls, and also that we may be able to measure the progress of the one by the nature and progress of the other.

Clinical study of the diseases of the walls of the heart teaches us that (1) the really serious effects of heart disease result from hypertrophy, or from dilatation, or from a combination of the two; (2) valvular diseases may exist up to the close of a long life without rendering the subject of them conscious of their presence; (3) it is possible to refer to cases in which, valve disease having existed without causing any inconvenience, something occurs which damages the condition of the muscular walls, and serious disturbance is the result; (4) there are cases in which some additional mischief occurs to valve disease already existing, and this mischief is remedied by the supervention of further compensatory hypertrophy; (5) the converse of the last class is seen in cases of valve disease in which the heart walls fail and the disease progresses; (6) cases occur in which valvular disease—i. e., incompetency—is caused by dilatation of the heart. These propositions show the primary importance which belongs to the study of diseases of the walls of the heart. As these walls are strong and efficient, or as they are weak, dilated, and inefficient, so will be the phenomena of disease with which we have to deal.

*Enlargement of the heart* is the most important of its pathological states, both from the frequency of its occurrence, and from the marked influence which it has over many other morbid conditions of the body. It is rarely the result of a single process, being usually due to a combination of hypertrophy of the principal elements which compose the cardiac walls, with dilatation of its cavities, the result of internal pressure.

The subject of hypertrophy of the heart seems to have been first noticed by Nicholas Massa, in 1534, who speaks of a heart "greatly enlarged." Our own Harvey, in 1601, described a heart very much enlarged occurring in a man "as the result of choler." Mayow, in 1681, describes a case in which, the left auriculo-ventricular orifice being obstructed, hypertrophy of both ventricles had occurred; and it would be difficult to give a truer or clearer description of our knowledge on the subject at the present day than that contained in his account. Vesalius, Senac, Corvisart, and Bertin, all described hypertrophy more or less accurately, but they gave it a subordinate place, regarding dilatation as the more important disease. Bertin first described hypertrophy fully, separating it from dilatation, and he formed a classification which is almost that which we use at the present day.

Enlargement of the walls of the heart may depend, first, on an increase in the muscular fibres; secondly, on an increase in the connective tissue; and thirdly, on an increase of fat.

The first form, *simple muscular hypertrophy*, is familiar enough in its character and appearances. The determination of the exact nature of the muscular change, whether by increased size or increased number of the fibres, or by both, is attended with so many difficulties that the question is still unsettled.

The second form, *connective tissue hypertrophy*, is one which has not yet received distinct and definite recognition. The disease first came under Dr. Quain's notice nearly twenty years ago, in a case recorded in the Pathological Transactions, vol. i. A similar change has been briefly alluded to by Corvisart, Laennec, Dr. Williams, Rokitansky, Sir W. Jenner, and Dr. Ormerod. Under the microscope a heart affected with this disease presents not only the usually limited amount of intermuscular fibrillar tissue and connective tissue cells, but a decided hyperplasia of this in the shape of connective tissue in all stages of development, from the globular to the spindle-shaped cell, and from this again to the bundle of fibrillæ. The muscular fibres are seen to be surrounded by this connective tissue, and more or less compressed by it. In many of the fibres granular and slightly fatty degeneration may be observed. In other parts of the heart the muscular fibres may be normal in appearance. The thickness of the walls is increased, as in simple hypertrophy, but their density and consistence is strikingly increased to a firm, tough, leathery character. When divided, the cut edges do not collapse, but remain stiffly prominent. In color, such a heart may vary from pale buff to deep purple, according to the amount of connective tissue and of blood present. But the change often occurs with less marked characters, and the hearts thus affected may not differ greatly from those hypertrophied by simple addition to the muscular fibres. This is doubtless the reason why the disease has been so often overlooked. The origin of this change is unquestionably to be sought for in a chronic interstitial inflammation or hyperplasia.

The third form, *hypertrophy of fat*, has usually been confounded with fatty degeneration. Where this change is moderate in amount, simple striæ of yellow tissue may be observed to lie amongst the fibres, and small masses of fat may appear beneath the endocardium. In the more extreme cases the muscular fibres may be almost concealed, and the columnæ carneæ may seem to arise from a mass of fat, as they are described to do by Laennec and Bizot. Under the microscope the fat is contained in cells as in other parts of the body. It is most abundant towards the outer surface. There, few muscular fibres can be seen, and the wide intervals between them are occupied by the fat-cells. Proceeding inwards, the fibres are more evident and the fat-cells fewer; and, finally, beneath the endocardium we have only a few cells lying here and there between the fibres. Even though overwhelmed with fat the fibres may still retain their organization; but, in all cases, their course and direction is more or less modified and distorted.

*Dilatation of the heart.* Its character and ordinary mode of origin are well known. It occurs in especial relation to degenerative processes in the cardiac wall, and particularly when occurring in the recently developed muscular fibre of commencing hypertrophy. Both processes continuing, we get those enormous examples of dilated hypertrophy sometimes seen, in some of which the heart reaches the weight of 46 ounces.

The *causes* of enlargement of the heart may be classified thus: (1) Agencies acting through the nervous system; (2) Agencies acting mechanically; (3) Agencies originating in disordered conditions of its nutritive functions.

Concerning the first of these causes Corvisart called attention to the increased frequency with which organic affections of the heart were met with during the French Revolution. In the overstrained excitement of our own more peaceful age we may recognize a cause equally efficient.

Statistics furnished by Dr. Farr clearly show two things: first, as an absolute fact, that during the last twenty years the total of deaths of males at all ages from heart disease have been more than doubled. Corrected for increase of population, the tables show that per 1,000 living between 1851 and 1855, .725 died from this cause, as compared with 1.085 from 1866 to 1870. Secondly, that during the years 1851—1870 the percentage of deaths from heart disease has remained quite unchanged in males under twenty, showing that the difference cannot be due to improved diagnosis of the disease, whereas the percentage in each succeeding period of twenty-five years has risen half as much again. Nevertheless, the same statistics show that there is almost no rise in the percentage of deaths of females from cardiac disease during the twenty-five years of life from twenty to forty-five.

The mechanical causes of hypertrophy are such as interfere with the free circulation of the blood. Severe muscular exertion, often repeated, is a frequent cause. The influence on the heart is, for the time, very distressing, and it may lead to permanent injury. Athletic sports in excess often do harm in the same way. The cardiac hypertrophy in Master Magrath was more likely the result than the cause of his sustained muscular efforts. No doubt to the influence of physical labor is due the far greater liability of men than women to cardiac hypertrophy. According to Dr. Vanderbyl, out of 40 cases 34 were in males, with an average weight 22½ oz., whilst only 6 were in females, with an average weight of 17½ oz.

A more frequently recognized class of mechanical causes consists of those occurring in the direct course of the circulation, in the heart itself, or in the vessels—e. g., contraction of the orifices of the heart, atheroma in the great vessels, constriction of the aorta, the distal obstruction in Bright's disease, and pregnancy. The influence of obstruction in the minute vessels in causing hypertrophy was pointed out by Mr. James, of Exeter, so far back as 1817, and afterwards more fully enunciated by Dr. Bright in his paper on albuminuria, published in 1836. Both he, and subsequently Dr. Snow, referred the increased action to a difficulty in sending the altered blood through the minute parts of the circulation; and Dr. Johnson has shown how this difficulty is produced by the increased contraction of the muscular walls of the small arteries, and subsequent hypertrophy. Traube imagined that the obstruction in the kidneys would explain the phenomenon.

The influence of pregnancy in causing hypertrophy has attracted less attention than it deserves. In 1826 Lacher announced, as the result of an examination of 130 healthy women who died in childbirth, that the heart was enlarged in pregnancy, the hypertrophy being confined to the left ventricle, whose walls were increased from a quarter to a third. The occurrence of the hypertrophy has since been amply confirmed. It is due, no doubt, to the necessity for an increased supply of blood to the womb, as well as to the mechanical resistance offered by the pressure of the gravid uterus upon the great vessels of the abdomen. Some considered that the albuminuria which occurs in pregnancy contributes in some measure to the cardiac enlargement, while most think with Traube that the latter exercises a causative influence upon the albuminuria.

Enlargement of the heart from the third group of causes—disordered conditions of the nutritive functions—is more frequently of a passive and degenerative kind. In chlorosis and anæmia the heart labors rapidly but ineffectually to send its diluted contents for the nourishment, imperfect though it be, of the system In such cases we see dilatation of the degenerated walls. In those again who live well and take little exercise, the heart becomes large, flabby, and infiltrated with fat. In Bright's disease some of the hypertrophy may be due to excited action caused immediately by the blood-state.

Of nutritive influences acting locally, one of the most interesting is the influence of pericarditis and adhesion. There has been much discussion concerning the state of the heart under these circumstances, some asserting that it is enlarged, others that it is diminished in size. As a general rule it is enlarged, but the enlargement is usually due to hypertrophy of the connective tissue. Such was the case in a heart described by Dr. Wilks in the "Pathological Transactions," vol. viii, in which one-half of the true muscular substance was replaced by fibrous tissue. Where, in these cases, the size is diminished, it is probably due to infiltration by a denser fibroid tissue—a cirrhotic process analogous to cirrhosis elsewhere.

Passing over the well-known causes of dilatation, there occur, lastly, cases of cardiac enlargement which cannot be referred to any one cause. Cases have been recorded by several observers. In some the dimensions of the heart have been enormous. It seems probable that such enlargement, without evident cause, may be due to fibroid infiltration. This view has been confirmed by an examination which has been made of one of these large hearts weighing 40½ oz., which has been for the last thirty years in the museum of St. George's Hospital. Under the microscope it was found to depend partly on hypertrophy of the muscular fibre, but also in great measure on a large increase in the amount of connective tissue.

---

The *local* effects of enlargement of the heart are sufficiently familiar. In considering its *systemic* effects it is well to bear in mind the three varieties of hypertrophy described in a previous lecture. In *simple muscular* hypertrophy, developed to overcome an obstacle, so long as the power is merely spent in overcoming the resistance, and is sufficient for that purpose, all goes well. But in some cases it is expended in other and in injurious directions. In distal obstruction, for instance, the intervening vessels are exposed to all the increased pressure from the powerfully acting ventricle, and various congestions result, controllable to some but only a limited extent by the vaso-motor nerves: if the vessels be sound, transudation may occur, as in dropsy; if rotten, they may give way, as seen in many cases of cerebral hemorrhage. Moreover, in the heart itself the increased power may be misdirected. In mitral regurgitation, the ventricular hypertrophy, unless counteracted by that in the auricle, may tend to increase the mischief in the lungs. In *connective tissue* hypertrophy the freedom of contraction of the fibres is restrained by the surrounding new tissue, and such a heart is dynamically weak. It would seem, so far as present observation goes, less likely to dilate than the simply degenerated heart. In the *fatty* hypertrophy there is a languid and feeble circulation, a sense of uneasiness and oppression at the chest, and embarrassed breathing, especially on an effort.

Dilatation of the heart, if confined to the left ventricle, causes passive congestion with all its results. When the right side is affected, these are conspicuously seen in the systemic circulation. Interference with the functions of nearly all the organs of the body, and general dropsy, result. Usually these conditions exist in conjunction—dilatation with some degree or form of hypertrophy; and the clinical physician has to unravel as best he can the tangled web before him.

Certain special effects of enlargement of the heart deserve attention. Its connection with cerebral hemorrhage has been a source of much difference of opinion among pathologists; some asserting, others denying, a causative relation. Dr. Burrows has shown a very close connection between apoplexy and disease of the heart, having ascertained their coexistence in three-fifths of the cases he investigated. Sir Thomas Watson doubts the influence of hypertrophy of the left ventricle in causing cerebral hemorrhage; while Eulenberg believes that it has such an influence only when it is dependent on peripheral disturbance of the circulation. In 65 autopsies in death by apoplexy, the accounts of which the lecturer has collected, the heart was increased in size without disease in 31, increased in size with valve disease in 12, and in 22 it was normal in size. Of the 31 cases without valve disease, the cerebral vessels were diseased in 17, and only in 4 of the 12 cases with valve disease. In the 22 in which the heart was normal, the vessels were diseased in 14. Moreover, examining the particular chambers affected, we find that in the 31 cases without valve disease the left ventricle was hypertrophied 29 times; in the 12 cases with valve disease it was hypertrophied 10 times. Out of the 65 cases, the left ventricle was hypertrophied in all 39 times. In the whole number of cases the kidneys were granular and contracted in 26, in 21 of which the left ventricle was hypertrophied. Hence we may conclude that in a given number of cases of apoplexy the enlarged heart is more frequently present than diseased cerebral vessels, nearly in the proportion of 4 to 3. Further, that in a given number of cases of apoplexy in which the heart is enlarged, the cerebral vessels are found as frequently healthy as diseased. Thirdly, that apoplexy is more frequently found with hypertrophy of the heart without than with valve disease in the proportion of 5 to 3. Lastly, that when the heart is enlarged, and the vessels of the brain diseased, cerebral apoplexy is found more frequently without than with valve disease in the proportion of 3 to 2.

*The relation of heart disease to phthisis.* First, as regards the size of the heart. In examining the records of 171 cases, it was enlarged in 25.66 per cent. of the male cases, and in only 7 per cent. of the female. In the males it was small in 53 per cent.; in females it was small in 67 per cent. In males it was normal in 21 per cent.; in females in 26 per cent. Some very small hearts were met with; in a girl, aged eight, the heart weighed only 2 oz.; in another, aged fourteen, only 1 oz. 14 drachms.

A second point was the duration of life in cases where the heart was hypertrophied. In 215 cases of phthisis used as a standard of comparison, in which no heart disease existed; 41.4 per cent. died under one year, 26 per cent. under two years, 19.5 per cent. under four years; 13 per cent. lived over four years. In 77 cases in which the heart was hypertrophied and dilated, 26 per cent. died under one year, 50.6 per cent. under two years, 6.5 under four years; 16.8 lived over four years. Thus we see that hypertrophy of the heart tends to prolong life in phthisis, and the result is most marked in the first and third years.

A third point was the relation of heart disease to hæmoptysis. In 80 cases in which the heart was hypertrophied (in 30 of which valve disease also existed), hæmoptysis occurred in 47 cases, or in 57.3 per cent. In 1381 cases of phthisis without heart disease, hæmoptysis occurred in 870, or in 63 per cent. Thus hypertrophy appears to have no influence in promoting hæmoptysis.

*The relation of heart disease to renal disease.* In 785 cases of heart disease, collected by Dr. Chambers, there was renal disease in 34 per cent. Hence a person with heart disease would be half as likely to have Bright's disease as not. Traube asserts that the renal congestion rarely ends in true Bright's disease, but the lecturer adheres to the opinion which he expressed twenty years ago—that the organic alterations very commonly result, both tubular and intertubular.

*Diagnosis of the nature of the cardiac enlargement.* The characters of the simple muscular hypertrophy are well known. Those of the second variety, the connective-tissue hypertrophy, are a strong heaving impulse, with a dull and obscure first sound. The signs are those of increased strength, but the increased strength is spent in great measure in overcoming the restricting action of the fibrous tissue which surrounds the muscular fibres. Hence there is evidence of circulatory weakness in the system generally. Such symptoms were present in the case of Dr. Hyde Salter, before alluded to. The fatty hypertrophy may be distinguished from that with which it is most likely to be confounded, as Dr. Shapter has pointed out, by the sharpness of the sounds, and by the general state of the patient.

Dilatation, the other great factor in producing cardiac enlargement, is evidenced by a peculiar square shape of the cardiac dullness, and short, clear and sharp cardiac sounds, accompanied, when the auriculo-ventricular openings have become dilated, with a systolic murmur, in the absence of any valve disease. By the presence, therefore, of concomitant dilatation, healthy muscular hypertrophy becomes considerably modified. But it is especially with degenerated hypertrophy that dilatation is combined. Hence the signs of the two conditions, dilatation and weakness, have come to be confounded, and the local phenomena of each, as well as their general effects, really due to the presence of an increased amount of connective tissue, or of fat, or of degeneration in the walls of the heart, have been attributed to the existence of dilatation alone. Hence the paramount importance of determining the textural state of the cardiac walls. In doing this, the sphygmograph and cardiograph both render assistance; the former, as Dr. Sanderson has shown, by indicating the arterial tension and the efficiency of the heart to do its work, while the latter indicates with great accuracy the duration of the systolic effort in the manner described by Mr. Garrod.

The treatment of enlargement of the heart has been much ruled—as so many other things in life, and it may even be said in death, have been—by fashion. In the time of Valsalva and Albertini, enlarged hearts were treated by systems so depleting and lowering that it was a question whether atrophy was not a frequent result of the treatment for hypertrophy. Now, stimulants and tonics are the favorite remedies. It is under such circumstances that we hear of the "uncertainty of remedies," of want of faith in physic, or of the still more hopeless doctrine that there are few or no diseases that can be cured. The word "cure" is a relative term. If it is intended to mean that an organ or texture destroyed by disease can be restored to its former condition, the list of incurable diseases may, it is true, be made a long one. But if, on the other hand, it is meant that the progress of an organic disease can in many instances be arrested, and the patient's health so far restored that the disease ceases to inconvenience him, then the list of curable diseases is each day becoming lengthened. Are phthisis and heart disease the incurable diseases they were once? Formerly they were not recognized till they had assumed an almost certainly fatal character. Now, detected and treated in an early stage, scores of cases do well, whether we owe the result to nature or not. If our knowledge were greater, both of the nature of pathological processes and of the action of medicines, our faith in physic would be stronger, because better founded. For example, if either the lowering or tonic treatment be applied indiscriminately to each of the three forms of cardiac hypertrophy, the ambiguous results need not surprise us. We may anticipate, as we understand them better, and learn the action of medicines upon them, that our results will be far more satisfactory.

In simple muscular hypertrophy, the physician has to discover its causes, and remove them as far as he can, and secure to the laboring organ all possible conditions of repose. We must try to subdue abnormally excited action, and, on the other hand, prevent the decay and degeneration which so often follow in the course of excessive development. In the action of drugs which have an influence in reducing the force of the heart—as aconite—lies a very promising field of investigation. In connective-tissue hypertrophy, if we could diagnose it in its early stage, remedies likely to subdue the inflammatory state in which it takes its origin might have a beneficial influence. In the third form—fatty hypertrophy—our treatment must be such as is calculated generally to prevent the formation of fat. In dilatation and dilated hypertrophy, the different conditions that are present demand corresponding measures for their relief. The dilating pressure would evidently be most immediately relieved by withdrawal of some of the blood, and this sometimes in urgent cases is very beneficial; but more frequently we attain the end by indirect measures—by purgatives and stimulation of the excretory organs, which, at the same time, diminish its volume and purify the blood. The next object must be to secure, if possible, compensatory hypertrophy by improving the nutrition generally, and especially by influencing the nutrition of the contractile substance of the heart. Two drugs especially have great power for this purpose, iron and digitalis. To prescribe the former before local congestions have been cleared away, is worse than useless; but after the blood stasis has been relieved, steel is well borne, and its effects are most valuable. The influence of digitalis has been most ably studied by many investigators, and especially of late by Drs. Brunton and Fothergill. There seems to be no doubt that it is a stimulant to the nervous ganglia of the heart, and neither a sedative to those nor a paralyzer of the vagus, as was once believed. Its administration is at once followed by better and more complete ventricular contraction. Dilatation is lessened, and with it all the local disturbances. The heart is enabled to supply all the organs with their due amount of blood, and there is a general restoration of healthy nutrition. The treatment, however, must be always secondary to the application of the eliminating agencies.

